# Implementing and Sustaining Family Care Programs in Real-World Settings: Barriers and Facilitators

**DOI:** 10.1093/geroni/igab046.1035

**Published:** 2021-12-17

**Authors:** Nancy Hodgson

**Affiliations:** University of Pennsylvania, School of Nursing, philadelphia, Pennsylvania, United States

## Abstract

This presentation will summarize the extant published studies on the translation of proven family care programs for dementia in different care settings. This review is the first to our knowledge to examine the specific implementation efforts deployed in care settings for different family caregiver programs. In this review, we sought to answer three basic questions: (1) What theory base(s) or conceptual framework(s) guided the implementation of evidence-based family care programs?; (2) What implementation strategies were used to support translation into practice?; and (3) What were the identified drivers of and barriers to organizational change required for adoption of an evidence-based program? Understanding the frameworks and strategies deployed in translational studies published to date can help guide future translation efforts, inform the design of new family caregiver support programs that optimize their implementation potential, and ultimately help to minimize the “family care gap.”

